# Altered Serum Uric Acid Levels in Kidney Disorders

**DOI:** 10.3390/life12111891

**Published:** 2022-11-15

**Authors:** Gheun-Ho Kim, Jae-Bum Jun

**Affiliations:** 1 Department of Internal Medicine, Hanyang University College of Medicine, Seoul 04763, Republic of Korea; 2 Department of Rheumatology, Hanyang University Hospital for Rheumatic Diseases, Seoul 04763, Republic of Korea

**Keywords:** autosomal dominant tubulointerstitial kidney disease, chronic kidney disease, COVID-19, Fanconi syndrome, hyperuricemia, hyponatremia, hypouricemia

## Abstract

Serum uric acid levels are altered by kidney disorders because the kidneys play a dominant role in uric acid excretion. Here, major kidney disorders which accompany hyperuricemia or hypouricemia, including their pathophysiology, are discussed. Chronic kidney disease (CKD) and hyperuricemia are frequently associated, but recent clinical trials have not supported the pathogenic roles of hyperuricemia in CKD incidence and progression. Diabetes mellitus (DM) is often associated with hyperuricemia, and hyperuricemia may be associated with an increased risk of diabetic kidney disease in patients with type 2 DM. Sodium-glucose cotransporter 2 inhibitors have a uricosuric effect and can relieve hyperuricemia in DM. Autosomal dominant tubulointerstitial kidney disease (ADTKD) is an important hereditary kidney disease, mainly caused by mutations of uromodulin (UMOD) or mucin-1 (MUC-1). Hyperuricemia and gout are the major clinical manifestations of ADTKD-UMOD and ADTKD-MUC1. Renal hypouricemia is caused by URAT1 or GLUT9 loss-of-function mutations and renders patients susceptible to exercise-induced acute kidney injury, probably because of excessive urinary uric acid excretion. Hypouricemia derived from renal uric acid wasting is a component of Fanconi syndrome, which can be hereditary or acquired. During treatment for human immunodeficiency virus, hepatitis B or cytomegalovirus, tenofovir, adefovir, and cidofovir may cause drug-induced renal Fanconi syndrome. In coronavirus disease 2019, hypouricemia due to proximal tubular injury is related to disease severity, including respiratory failure. Finally, serum uric acid and the fractional excretion of uric acid are indicative of plasma volume status; hyperuricemia caused by the enhanced uric acid reabsorption can be induced by volume depletion, and hypouricemia caused by an increased fractional excretion of uric acid is the characteristic finding in syndromes of inappropriate anti-diuresis, cerebral/renal salt wasting, and thiazide-induced hyponatremia. Molecular mechanisms by which uric acid transport is dysregulated in volume or water balance disorders need to be investigated.

## 1. Introduction

Uric acid is the end-product of purine metabolism in humans and apes, unlike other mammals which have uricase. This genetic evolution has led humans to exhibit plasma uric acid levels that are 3–10 times higher than those of other mammals [1]. We recently analyzed data from the Korean Genome and Epidemiology Study (which enrolled 58,981 men and 113,989 women) and found that the mean serum uric acid level was higher in men (5.7 mg/dL) than in women (4.2 mg/dL). When hyperuricemia and hypouricemia were defined as serum uric acid levels > 7 mg/dL and ≤2.0 mg/dL, respectively, their prevalence rates differed according to age and sex. Noticeably, the prevalence of hyperuricemia increased with aging in women but not in men. Overall, the prevalence rates of hyperuricemia in men and women were 133 and 8 per 1000 people, respectively, while the prevalence rates of hypouricemia in men and women were 1 and 6 per 1000 people, respectively [2]. Estrogen may play a protective role in hyperuricemia by regulating the activity of uric acid transporters in the kidney [3]. This enhances the renal clearance of uric acid and may be linked to decreased cardiovascular risk. These altered serum uric acid levels may have clinical impacts. In a large cohort study of Korean men and women, both low and high uric acid levels were associated with an increased mortality rate, supporting a J-shaped relationship between serum uric acid and adverse clinical outcomes [4].

Serum uric acid levels are normally maintained by the balance between hepatic production and renal and intestinal excretion. Purines, derived from diet (100–200 mg/day) and the cellular metabolism of nucleic acids (500–600 mg/day), are metabolized to uric acid in the liver. The final step of uric acid synthesis (700 mg/day) is catalyzed by the enzyme xanthine oxidase, and xanthine oxidase inhibitors, such as allopurinol and febuxostat, are clinically useful in lowering uric acid. The kidneys play a dominant role in uric acid excretion because they excrete approximately 70% of the uric acid produced daily (500 mg/day), while the remaining 30% (200 mg/day) is excreted by the intestine [5].

**Figure 1:**
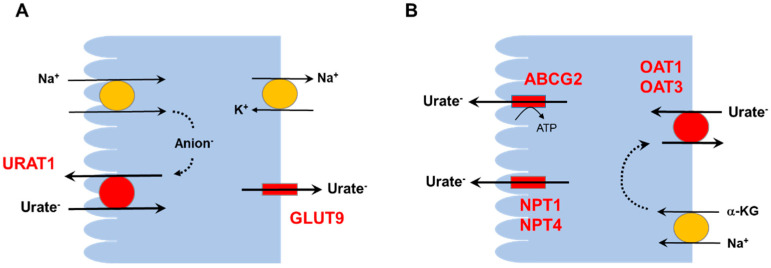
Major transporters for uric acid reabsorption and secretion in the proximal tubule. (**A**): Na^+^-dependent anion transport increases intracellular concentrations of anions that exchange with luminal urate by URAT1. GLUT9 acts as the basolateral exit for urate reabsorption. (**B**): OAT1 and OAT3 transport urate through the basolateral membrane in exchange with α-KG. At the apical membrane, urate is secreted via ABCG2, NPT1, and/or NPT4. Abbreviations: ABCG2, ATP-binding cassette subfamily G member 2; GLUT9, glucose transporter 9; α-KG, α-ketoglutarate; NPT1, sodium-phosphate cotransporter 1; NPT4, sodium-phosphate cotransporter 4; OAT1, organic anion transporter 1; OAT3, organic anion transporter 3; SGLT2, sodium-glucose cotransporter 2; URAT1, urate transporter 1.

Normally, 90% of glomerular-filtered uric acid is reabsorbed by the proximal tubule. However, uric acid transport in the proximal tubule is bidirectional (Figure 1); reabsorption is mainly mediated by apically located urate transporter 1 (URAT1) and basolaterally located glucose transporter 9 (GLUT9), and secretion is exerted by basolaterally located organic anion transporters 1 and 3 (OAT1, OAT3) and apically located ATP-binding cassette superfamily G member 2 (ABCG2) and sodium-dependent, inorganic phosphate transporters 1 and 4 (NPT1, NPT4). Considering the usual ranges of the fractional excretion of uric acid, the transporters that mediate uric acid reabsorption may be more influential than those mediating uric acid secretion [6]. Most uricosuric agents, such as benzbromarone, probenecid, and lesinurad, inhibit URAT1 [7].

Among the angiotensin II receptor blockers, losartan has uricosuric action. In hypertensive patients, losartan decreases serum uric acid in association with a concomitant increase in urinary uric acid excretion. However, losartan does not affect serum and urine uric acid levels in hypertensive patients with a loss-of-function mutation of URAT1 [8], suggesting that losartan inhibits URAT1 in the proximal tubule. The uricosuric action of losartan may be affected by URAT1 gene polymorphisms [9,10].

Declines in the glomerular filtration rate (GFR) and/or the dysregulation of the proximal tubular transport can lead to altered serum uric acid levels. In this paper, the associations between kidney disorders and altered serum uric acid levels are discussed. Both hyperuricemia and hypouricemia are frequently found in primary or secondary kidney diseases (Table 1). Chronic kidney disease (CKD) is commonly associated with primary or secondary hyperuricemia. Diabetes mellitus (DM) is often accompanied by hyperuricemia, irrespective of kidney dysfunction. Hyperuricemia is an important clinical presentation of autosomal dominant tubulointerstitial kidney disease (ADTKD), a rare genetic cause of CKD. Other hereditary or acquired proximal tubular defects can present with hypouricemia. While hyperuricemia may be associated with volume depletion, hypouricemia is a characteristic finding in syndromes of inappropriate antidiuresis (SIAD), cerebral/renal salt wasting (CSW/RSW), and thiazide-induced hyponatremia. This review focuses on the role of the kidneys in the pathogenesis of hyperuricemia and hypouricemia, but primary gout and uric acid nephropathy will not be covered. In gout, chronic gouty nephropathy is an important cause of chronic tubulointerstitial nephritis, causing CKD. In tumor lysis syndrome, acute uric acid nephropathy can be complicated, leading to acute kidney injury (AKI).

## 2. Hyperuricemia and the Kidneys

**Figure 2:**
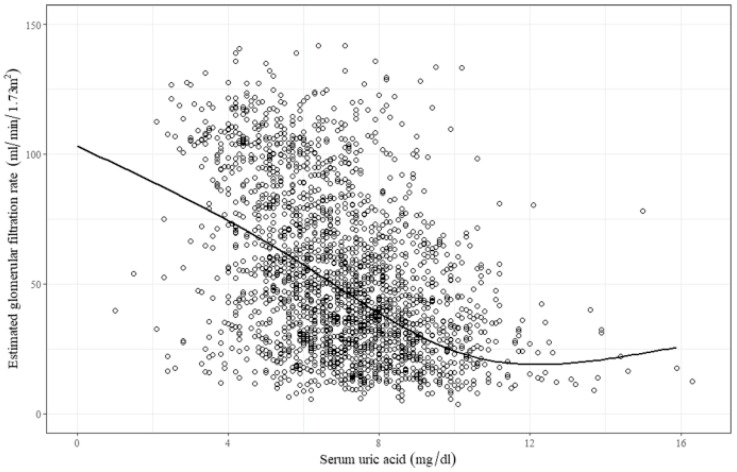
Serum uric acid levels according to changes in eGFR. As eGFR decreases in patients with chronic kidney disease, the serum uric acid level tends to increase. Abbreviation: eGFR, estimated glomerular filtration rate. Adapted from the article by Oh et al. [12] according to the Creative Commons Attribution 4.0 International License.

Considering the solubility of uric acid, hyperuricemia can be defined as a serum uric acid level > 7.0 mg/dL, regardless of sex [11]. Because the kidneys are the major route of uric acid excretion, a reduction in GFR may lead to hyperuricemia. Figure 2 shows a correlation between the estimated GFR (eGFR) and serum uric acid level in a Korean cohort [12]. An increase in the proximal tubular reabsorption of uric acid can also produce hyperuricemia.

The association of hyperuricemia with increased cardiovascular risk may partly be explained by the activation of the renin-angiotensin system (RAS). When mild hyperuricemia was induced in rats by providing oxonic acid in the diet, blood pressure was elevated, and juxtaglomerular renin expression increased [13]. Plasma renin activity and plasma aldosterone concentration were also elevated in rats with hyperuricemia [14], but these associations were unclear in adults with essential hypertension [15]. Intrarenal RAS activity may be affected by hyperuricemia in humans [16].

### 2.1. Asymptomatic Hyperuricemia in CKD

A systematic review and meta-analysis recently reported that higher uric acid levels increased the risk of CKD incidence and progression [17]. However, whether to treat or not to treat asymptomatic hyperuricemia in CKD patients is still under debate. Two questions need to be answered regarding the pathogenic role of uric acid in CKD. First, does hyperuricemia accelerate the progression of CKD? Two recent randomized controlled clinical trials reported negative results for this question [18]. In the CKD-FIX trial, 363 patients with stage 3 or 4 CKD and no history of gout were randomized to receive allopurinol (100–300 mg/day) or placebo for approximately 1.5 years. Despite the lowering of serum uric acid from 8.2 mg/dL to 5.1 mg/dL, allopurinol treatment did not significantly slow a decline in eGFR compared to the placebo [19]. The PERL trial studied 530 patients with type 1 diabetes, evidence of kidney disease (eGFR, 40–100 mL/min/1.73 m^2^), and serum uric acid levels > 4.5 mg/dL. Participants received either allopurinol (100–400 mg/day) or placebo for 3 years. Despite the lowering of serum uric acid from 6.1 mg/dL to 3.7 mg/dL, the average annual loss in measured GFR did not differ between the two groups [20]. Although these studies had high dropout rates (20–30%), the results were compatible with a Mendelian randomization study [21], suggesting that uric acid does not predict CKD progression. The outcomes might have been different if the mean baseline uric acid levels had been higher, e.g., approximately 10 mg/dL.

Interestingly, febuxostat might have some favorable effects on renal progression. Febuxostat slowed a decline in eGFR over 6 months in 45 patients with CKD stages 3 and 4, compared to 48 in the placebo group [22]. In cardiac surgery patients with an eGFR ≤ 60 mL/min/1.73 m^2^, kidney function was more preserved by febuxostat than by allopurinol [23]. Larger clinical trials are necessary to compare the effects between febuxostat and allopurinol.

The next question to consider is whether hyperuricemia increases the incidence of CKD. Observational studies consistently report that hyperuricemia predicts the development of CKD [24]. However, the pathogenic role of uric acid in new-onset CKD is unclear. Hassan et al. recently examined the association between uric-acid-lowering therapy and the incidence of CKD in a large cohort of U.S. veterans with no pre-existing CKD and found that, in patients with kidney function within the reference range eGFR > 60 mL/min/1.73 m^2^, uric-acid-lowering therapy was not associated with the preservation of kidney function. In patients with baseline serum uric acid levels of ≤8 mg/dL, uric-acid-lowering therapy was paradoxically associated with a higher risk of incident CKD, indicated by the development of an eGFR < 60 mL/min/1.73 m^2^ or new-onset albuminuria [25]. Therefore, evidence for the causal effect of uric acid on CKD is currently lacking. Properly powered randomized clinical trials in patients with no pre-existing CKD are necessary.

### 2.2. Diabetic Kidney Disease (DKD)

Hyperuricemia is often associated with diabetes mellitus (DM) [11], and it seems that insulin resistance correlates with serum uric acid levels and inversely correlates with the renal clearance of uric acid [26]. In addition, hyperuricemia may be associated with an increased risk of DKD in patients with type 2 DM [27]. However, the causal relationship between hyperuricemia and DM or DKD has not been documented.

Sodium-glucose cotransporter 2 (SGLT2) inhibitors are first-line therapeutics in DKD because of their cardiorenal protection. They have a uric-acid–lowering effect, which was confirmed by a meta-analysis of 62 clinical trials [28]. Treatment with an SGLT2 inhibitor consistently lowered serum uric acid concentrations in a total of 34,941 patients with type 2 DM. When baseline uric acid levels were within the normal limit, the SGLT2 inhibitors typically decreased serum uric acid concentrations by 0.60–0.75 mg/dL in trials lasting 6–12 months. This uric acid–lowering effect was rapidly induced within days and persisted throughout trials of a 2-year duration [29].

**Figure 3:**
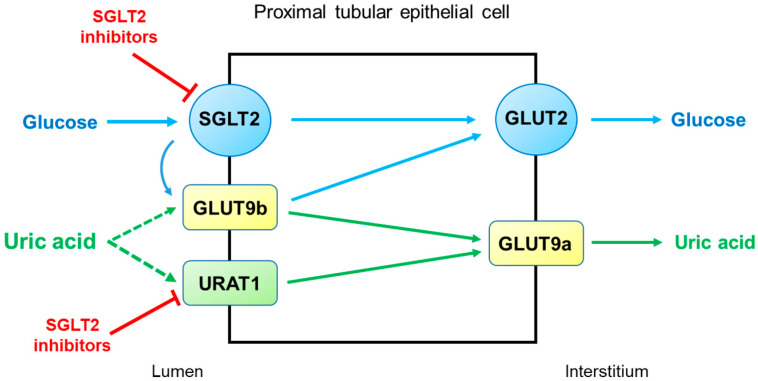
Potential mechanisms by which SGLT2 inhibitors increase uricosuria in the proximal tubule. URAT1 and GLUT9 are the major pathways for uric acid reabsorption. When SGLT2 is inhibited by SGLT2 inhibitors, GLUT9b is overwhelmed by excessive glucose and its capacity for uric acid transport is diminished. SGLT2 inhibitors can also inhibit URAT1, further increasing uricosuria. Abbreviations: GLUT9, glucose transporter 9; SGLT2, sodium-glucose cotransporter 2; URAT1, urate transporter 1.

SGLT2 inhibitors reduce serum uric acid concentrations by elevating renal uric acid excretion. However, the molecular mechanisms by which SGLT2 inhibitors exert uricosuric action are unclear. It seems likely that SGLT2 inhibitors induce hyperuricosuria by reducing reabsorption rather than by enhancing the secretion of uric acid in the proximal tubule (Figure 3). As described above, URAT1 and GLUT9 are the major uric acid transporters for reabsorption. GLUT9 has two isoforms, GLUT9a and GLUT9b, which transport both glucose and uric acid in the proximal tubule [30]. Unlike GLUT9a, GLUT9b is presumed to be located in the apical membrane and is overwhelmed by excessive glucosuria when SGLT2 is inhibited, with its capacity to reabsorb uric acid markedly diminished [29]. On the other hand, the role of URAT1 in the uricosuric effect of SGLT2 inhibition was proposed in mice [31]. This was also suggested in humans because uricosuria induced by combination therapy with empagliflozin and benzbromarone did not differ from benzbromarone monotherapy [32]. Thus, URAT1 seems to be the target of SGLT2 inhibitors to induce hyperuricosuria.

### 2.3. ADTKD

ADTKD is a rare genetic kidney disease characterized by tubular damage and interstitial fibrosis that can progress to end-stage kidney disease. More accurately, ADTKD is a group of conditions with autosomal dominant inheritance. Mutations in UMOD and MUC1 are the typical causes of ADTKD, but other rarer (REN, SEC61A1), atypical (DNAJB11), or heterogeneous (HNF1B) subtypes have also been described [33]. UMOD encodes uromodulin, the most abundant protein secreted in normal urine, but with unidentified multiple roles in renal physiology and pathophysiology. MUC1 encodes transmembrane epithelial mucin-1 and has roles in epithelial barrier protection. REN encodes preprorenin, which is converted to prorenin and renin, and has a role in regulating blood pressure and electrolyte balance. HNF1B encodes homeodomain-containing transcription-factor hepatocyte nuclear factor 1β (HNF1β), which has a role in the development of several organs, including the kidneys. Because of increased awareness and genetic testing, ADTKD has become the third-most-common hereditary monogenic kidney disease in Western countries, after autosomal dominant polycystic kidney disease and type IV collagen mutations [34]. Until its recent definition as a distinct disease entity, the following various terms were used to describe this disorder: uromodulin kidney disease, uromodulin-associated kidney disease, mucin-1 kidney disease, familial juvenile hyperuricemic nephropathy, medullary cystic kidney disease type 1, medullary cystic kidney disease type 2, and renin-associated kidney disease. Because these terms were confusing or misleading, an international consensus by Kidney Disease Improving Global Outcomes (KDIGO) was reached to use the term “autosomal dominant tubulointerstitial kidney disease”, with a subclassification based on underlying genetic defects [34].

Typical ADTKD patients can present with asymptomatic azotemia incidentally found during routine laboratory testing [35]. This diagnosis should be suspected when slowly progressive CKD is accompanied by an absence of significant proteinuria with bland urine sediment, normal or slightly elevated blood pressure, normal- or small-sized kidneys, and a positive family history of progressive CKD [33,34]. A precise diagnosis can be made genetically, and a kidney biopsy is usually unnecessary because it shows non-specific interstitial fibrosis and tubular atrophy [35].

Notably, hyperuricemia and secondary gout are important clinical clues for the diagnosis of ADTKD. In patients with ADTKD-UMOD and ADTKD-REN, hyperuricemia frequently occurs in childhood, and early-onset gout can present during the teenage years with hypouricosuric hyperuricemia [34,36]. Although the molecular mechanisms by which hyperuricemia is induced are unclearly defined, proximal tubular adaptations are conceivable in response to plasma volume contractions induced by salt-losing nephropathy in ADTKD-UMOD and anemia and hypotension in ADTKD-REN. A compensatory response in the proximal tubule may accompany the enhanced reabsorption of uric acid and lead to hyperuricemia [37]. Aged uromodulin knockout mice, in addition to hyperuricemia and hypertension, had an upregulation of uric acid transporter URAT1 and sodium-hydrogen exchanger 3 (NHE3) in the proximal tubule [38].

In contrast, ADTKD-MUC1 has a prevalence of hyperuricemia and gout similar to those of other advanced kidney diseases [39]. As in other cases of CKD, allopurinol and febuxostat are indicated to lower serum uric acid levels. Hyperuricemia and hypomagnesemia are characteristic laboratory features in ADTKD-HNF1B. In particular, hyperuricemia was reported in 37% of pediatric patients with an early onset at infancy [40] and in 20% of adult patients [41]. The reason why hyperuricemia is more frequently reported in infants needs to be explained. Renal magnesium wasting in ADTKD-HNF1B may be related to the finding that transcription of FXYD2, the γ-subunit of the Na^+^/K^+^-ATPase, is regulated by HNF1β in the distal tubule [42]. In the distal convoluted tubule, transcellular Mg^2+^ uptake is driven by Na^+^/K^+^-ATPase activity, which controls the membrane potential at the apical membrane [34].

### 2.4. Thiazide and Loop Diuretics

Hyperuricemia is frequently complicated by diuretic use, and thiazide and loop diuretics are the major causes of secondary hyperuricemia [43]. Diuretic-induced hyperuricemia may occur within a few days after the initiation of medication, appears to be dose-dependent, and persists during the period of administration [43,44].

**Figure 4:**
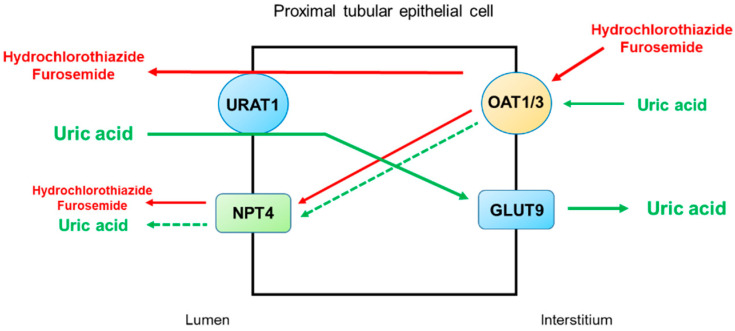
Potential mechanisms by which thiazide and loop diuretics reduce uricosuria in the proximal tubule. URAT1 is the major transporter for uric acid reabsorption, and OAT1 and NPT4 are the major pathways for uric acid secretion. Thiazide and loop diuretics enter the proximal tubular cell basolaterally via OAT1 and OAT3 and exit via apically located URAT1 or NPT4. Competitive binding between diuretics and uric acid for the basolateral OAT1 and OAT3 and apical NPT4 would reduce uric acid secretion by the proximal tubule. The apically located URAT1 acts as an anion exchanger and may reabsorb uric acid in exchange for the secretion of thiazide or loop diuretics. Thus, a decrease in uric acid secretion, as well as an increase in uric acid reabsorption by the proximal tubule, will lead to diuretic-induced hyperuricemia. Abbreviations: GLUT9, glucose transporter 9; NPT4, sodium-dependent, inorganic phosphate transporter 4; OAT1, organic anion transporter 1; OAT3, organic anion transporter 3; URAT1, urate transporter 1.

Thiazide and loop diuretics can directly and indirectly increase the reabsorption of uric acid in the proximal tubule. The direct interaction of diuretics with uric acid transporters for reabsorption or secretion may occur, and diuretic-induced volume depletion indirectly increases uric acid reabsorption by the proximal tubule [45]. Figure 4 illustrates the secretory pathways of thiazide and loop diuretics for delivery to the tubular sites of action and the potential inhibition or stimulation of uric acid transporters in the proximal tubule. Thiazide and loop diuretics enter the proximal tubular cell via basolaterally located organic anion transporters OAT1 and OAT3, and they may exit via apically located URAT1 or NPT4 [46]. On the other hand, URAT1 is the major transporter for uric acid reabsorption, and OAT1 and NPT4 are the major pathways for uric acid secretion. Therefore, competitive binding between diuretics and uric acid for the basolateral OAT1 and OAT3 and apical NPT4 would reduce uric acid secretion by the proximal tubule. The apically located URAT1 acts as an anion exchanger and may reabsorb uric acid in exchange for the secretion of thiazide or loop diuretics [47]. Thus, a reduction in uric acid secretion, as well as an increase in uric acid reabsorption by the proximal tubule, will lead to diuretic-induced hyperuricemia.

## 3. Hypouricemia and the Kidneys

Hypouricemia, defined as a serum uric acid level ≤ 2.0 mg/dL [11], can be produced by the renal wasting of uric acid. Considering normal values of the fractional excretion of uric acid (~10%), the essential role of the kidneys is closer to the conservation rather than elimination of uric acid. Thus, impaired uric-acid-transport function in the proximal tubule leads to hypouricemia. In many cases, hypouricemia can go unnoticed because it usually occurs without symptoms. Its clinical consequences need to be investigated.

### 3.1. Renal Hypouricemia

Hypouricemia caused by a renal tubular defect has been termed “renal hypouricemia”, and loss-of-function mutations of the *SLC22A12* and *SLC2A9* genes are called type 1 and type 2 renal hypouricemia, respectively [48]. The *SLC22A12* and *SLC2A9* genes encode the apically located URAT1 and the basolaterally located GLUT9, respectively, the main reabsorptive uric acid transporters in the proximal tubule. In renal hypouricemia type 1 or 2, the fractional excretion of uric acid increases to much higher than 10% despite a very low level of serum uric acid. Patients with renal hypouricemia can present with hematuria, urolithiasis, and exercise-induced AKI.

Among different mutations in the *SLC22A12* gene, W258X (rs121907892) was predominant in patients with type 1 renal hypouricemia in reports from Japan [49] and Korea [50]. While type 1 renal hypouricemia mainly occurs in Asian children, cases of type 2 renal hypouricemia were reported in various parts of the world, including Asia, the Middle East, and Europe. Patients with type 2 renal hypouricemia were often diagnosed during adulthood [51].

Exercise-induced AKI is an important clinical presentation of renal hypouricemia. It can be differentiated from rhabdomyolysis-associated AKI because of the absence of elevated creatinine kinase levels and myoglobinuria. Urine data are compatible with pre-renal azotemia, and kidney function gradually improves with hydration. The characteristic computed tomography findings are patchy renal vasoconstriction or multiple patchy wedge-shaped delayed-contrast enhancements in the kidneys [52]. The reason why exercise-induced AKI can occur in patients with renal hypouricemia is unclear. Two different aspects were viewed in the pathogenesis of renal injuries: a low serum uric acid level and a high urine uric acid level. Interestingly, uric acid may function as an antioxidant in plasma and can act as a pro-oxidant within the cell [53]. In patients with hypouricemia, the antioxidant activity of uric acid is overwhelmed by the massive concentration of reactive oxygen species produced by exhaustive exercise. Thus, the loss of antioxidant activity in plasma may lead to vascular constriction and endothelial damage, progressing to AKI [54]. According to the other viewpoint, in the kidneys of renal hypouricemia after strenuous exercise, intense inflammation might be stimulated by a high intraluminal concentration of uric acid in the proximal straight tubule and the thick ascending limb of Henle’s loop [55]. The nucleotide-binding oligomerization domain-like receptor family pyrin domain-containing 3 (NLRP3) inflammasome signal associated with exercise-induced AKI in URAT1-uricase double-knockout mice was attenuated by uric-acid-lowering therapy using allopurinol or topiroxostat [56].

### 3.2. Fanconi Syndrome

Fanconi syndrome is caused by generalized proximal tubular dysfunction and manifested by phosphaturia, renal glucosuria, aminoaciduria, tubular proteinuria, and proximal renal tubular acidosis [57]. It is secondary to systemic disease in many cases, and it is called renal Fanconi syndrome (RFS) when renal-limited. Because the proximal tubule is the only nephronal segment capable of handling uric acid in the kidneys, hypouricemia and hyperuricosuria are also important clues for the diagnosis of Fanconi syndrome.

The etiology of RFS includes inherited and acquired disorders and RFS, when diagnosed in adults, is most commonly associated with drug toxicity [58]. The frequently implicated agents include cisplatin, ifosfamide, tenofovir, sodium valproate, and aminoglycoside antibiotics [59]. When these drugs accumulate in the proximal tubular cells because of a traffic jam between the basolateral entrance and the apical exit, mitochondrial DNA depletion and dysfunction occur, which can ultimately cause a kind of proximal tubulopathy characterized by AKI and Fanconi syndrome [60].

With advances in molecular genetics, three genetic forms of RFS have been identified: Fanconi renotubular syndrome (FRTS) type 1, 2, and 3 [57]. These were previously considered to be idiopathic Fanconi syndrome, but cases of idiopathic adult-onset RFS are still being reported [61]. FRTS1 is inherited in an autosomal dominant fashion and associated with progressive kidney failure. The gene and gene product altered in FRTS1 have not been identified, but the gene locus for this disease was mapped to human chromosome 15q15.3 [62]. FRTS2 is characterized by phosphate wasting and rickets, and is caused by a mutation in *SLC34A1*, which encodes the phosphate transporter NaPi-IIa [63]. FRTS3 is the prototype of RFS characterized by no kidney failure [57] and the autosomal dominant inheritance of heterozygous missense mutation in the *EHHADH* gene [64].

### 3.3. Coronavirus Disease 2019 (COVID-19)

Kidney involvement in patients with COVID-19 is common and can range from urinary abnormalities to AKI requiring kidney replacement therapy. The COVID-19-associated AKI is associated with high mortality and serves as an independent risk factor for all-cause in-hospital death in patients with COVID-19 [65]. According to kidney biopsies and autopsy series, acute tubular injury is the dominant renal pathology, although glomerular pathologies, such as collapsing glomerulopathy and thrombotic microangiopathy, have been found [66].

Although kidney damage may result from hemodynamic factors and dysfunctional immune responses in patients with COVID-19 [67], there is also some evidence of a direct kidney infection caused by severe acute respiratory syndrome coronavirus 2 (SARS-CoV-2). SARS-CoV-2 was detected in the kidneys of patients with COVID-19 using immunohistochemistry, immunofluorescence, real-time reverse transcription–polymerase chain reaction, in situ hybridization, and electron microscopy [68]. Because the angiotensin-converting enzyme 2 receptor target of SARS-CoV-2 is highly expressed in proximal tubule cells, Werion et al. investigated specific manifestations of proximal tubule dysfunction in patients with COVID-19 [69]. In a cohort of 49 patients requiring hospitalization, low-molecular-weight proteinuria, neutral aminoaciduria, and the defective handling of uric acid or phosphate were found. Among these features of proximal tubule dysfunction, hypouricemia with inappropriate uricosuria was independently associated with disease severity and a significant increase in the risk of respiratory failure, necessitating invasive mechanical ventilation. The authors also documented prominent proximal tubular injury with brush border loss, acute tubular necrosis, intraluminal debris, and a marked decrease in the expression of megalin in the brush border. Particles resembling coronaviruses were identified in the proximal tubular cells by transmission electron microscopy [69].

These results were validated by two independent cohorts involving 192 and 325 patients hospitalized with COVID-19 in Brussels, Belgium [70]. The same conclusion was drawn that in COVID-19 patients requiring hospitalization, hypouricemia is common and associated with disease severity and progression to respiratory failure. Similar findings were reported from a pediatric patient [71] and a Chinese cohort involving 1854 patients [72].

### 3.4. Hyponatremic Disorders: SIAD, RSW, and Thiazide-Induced Hyponatremia

Several hyponatremic disorders are associated with altered serum uric acid levels. While the serum uric acid level is normal or elevated in hypovolemic hyponatremia, hypouricemia is typically associated with SIAD, CSW/RSW, and thiazide-induced hyponatremia.

#### 3.4.1. SIAD

Two supplemental features of water retention, serum uric acid < 4 mg/dL, and blood urea nitrogen (BUN) < 10 mg/dL, are very useful in the diagnosis of SIAD [73]. Hypouricemia in SIAD is the result of an increased uric acid clearance related to a reduction in proximal tubular uric acid reabsorption [74]. The correction of hyponatremia by water restriction normalizes uric acid clearance, despite the persistent inappropriate secretion of arginine vasopressin [75]. However, the mechanism of decreased tubular uric acid reabsorption in SIAD is unclear. In subjects with desmopressin-induced hyponatremia, the fractional excretion of uric acid was not elevated, unlike in hyponatremic patients with SIAD [76]. These results suggest a role of vasopressin V1 receptor (V1R) stimulation in the increase in renal uric acid clearance. Twenty years later, Taniguch et al. examined this hypothesis at the level of renal uric acid transporters [77] and showed that terlipressin-treated rats had a downregulation of GLUT9 (for uric acid reabsorption) and an upregulation of ABCG2 and NPT1 (for uric acid secretion) in association with hypouricemia and an increased fractional excretion of uric acid. It is also conceivable that the paracellular reabsorption of uric acid might be suppressed by V1R signaling in the proximal tubule.

#### 3.4.2. CSW/RSW

CSW was first described in 1950 from three patients presenting with hyponatremia, clinical evidence of volume depletion (e.g., hypotension, unexplained tachycardia, low central venous pressure, pre-renal azotemia, hemoconcentration, or metabolic alkalosis), and renal sodium wasting in the setting of various forms of cerebral disease [78]. Additionally, intracranial pathologies were presumed to disrupt efferent neural pathways to the kidneys, resulting in salt wasting and hypovolemia. However, the accurate assessment of volume status is clinically unavailable, and achieving a differential diagnosis between CSW and SIAD is difficult. Both conditions present with hyponatremia with a low plasma osmolality, an inappropriately elevated urine osmolality, a urine sodium concentration usually > 40 mmol/L, and a low serum uric acid concentration due to an increased fractional excretion of uric acid [79]. Thus, CSW patients have an unusual combination of hypovolemia and hypouricemia.

Controversy regarding the existence and prevalence of CSW remains [80]. Despite seven decades of investigation, the pathophysiologic basis for the natriuresis and uricosuria of CSW is not yet proven. The general belief is that CSW rarely occurs and may be a subtype of SIAD [81]. However, Maesaka et al. reported that CSW is not rare and more often occurs in patients without cerebral diseases than those with cerebral diseases [82], and they proposed a change in the terminology from CSW to RSW [83]. In addition, these authors emphasized the characteristics of CSW/RSW in comparison to SIAD; specifically, hypouricemia and an increased fractional excretion of uric acid persist following the correction of hyponatremia [84], and this proximal tubular defect is often accompanied by an increased fractional excretion of phosphate [85]. Prospective studies of larger groups of patients are necessary to confirm the diagnostic significance of these parameters.

#### 3.4.3. Thiazide-Induced Hyponatremia

As described above, hyperuricemia can be induced by the use of thiazide and loop diuretics. However, hypouricemia is a characteristic laboratory finding when hyponatremia is induced by thiazide diuretics. Previous studies have shown that patients with thiazide-induced hyponatremia have clinical features of SIADH, including a low serum uric acid concentration and a low BUN level [86], and the mechanism of thiazide-induced hyponatremia can be explained by nephrogenic antidiuresis [87]. How uric acid transport is disturbed by thiazide-induced renal water retention remains to be answered.

## 4. Conclusions

Serum uric acid levels are altered by changes in the renal excretion of uric acid. Although hyperuricemia and CKD are frequently associated, the pathogenic roles of hyperuricemia in CKD incidence and progression are still unclear. In DKD, SGLT2 inhibitors exert a uricosuric effect by inhibiting uric acid transporters in the proximal tubule. Hyperuricemia and gout are the major clinical manifestations in ADTKD, a kidney disease recently labeled as hereditary. Renal hypouricemia caused by URAT1 or GLUT9 loss-of-function mutations is susceptible to exercise-induced AKI, probably because of an excessive urinary excretion of uric acid. Hypouricemia derived from renal uric acid wasting is a component of Fanconi syndrome, which may be hereditary or acquired. Drug-induced renal Fanconi syndrome can be explained by mitochondrial injury in the proximal tubule. Hypouricemia is associated with proximal tubular injury in COVID-19 and is related to disease severity, including respiratory failure. Among hyponatremic disorders, hypouricemia is a characteristic laboratory finding in SIAD, CSW/RSW, and thiazide-induced hyponatremia. The molecular mechanisms by which uric acid transport is dysregulated in volume or water balance disorders need to be clarified.

## Figures and Tables

**Table 1:** Major kidney disorders that accompany altered serum uric acid levels.

Hyperuricemia
Chronic kidney disease (CKD)
Diabetes mellitus and diabetic kidney disease (DKD)
Autosomal dominant tubulointerstitial kidney disease (ADTKD)
Thiazide and loop diuretics
Hypouricemia
Renal hypouricemia type 1 and type 2
Fanconi syndrome: inherited or acquired
Coronavirus disease 2019 (COVID-19)
Hyponatremic disorders
Syndrome of inappropriate antidiuresis (SIAD)
Cerebral/renal salt wasting
Thiazide-induced hyponatremia

## Data Availability

Not applicable.
